# Aflatoxins Contamination in Feed Commodities: From Occurrence and Toxicity to Recent Advances in Analytical Methods and Detoxification

**DOI:** 10.3390/microorganisms11102614

**Published:** 2023-10-23

**Authors:** Slim Smaoui, Teresa D’Amore, Maria Tarapoulouzi, Sofia Agriopoulou, Theodoros Varzakas

**Affiliations:** 1 Laboratory of Microbial, Enzymatic Biotechnology and Biomolecules (LBMEB), Center of Biotechnology of Sfax, University of Sfax-Tunisia, Sfax 3029, Tunisia; 2 IRCCS CROB, Centro di Riferimento Oncologico della Basilicata, 85028 Rionero in Vulture, Italy; teresa.damore@crob.it; 3 Department of Chemistry, Faculty of Pure and Applied Science, University of Cyprus, P.O. Box 20537, Nicosia CY-1678, Cyprus; tarapoulouzi.maria@ucy.ac.cy; 4 Department of Food Science and Technology, University of the Peloponnese, Antikalamos, 24100 Kalamata, Greece; s.agriopoulou@uop.gr

**Keywords:** aflatoxins, feed matrices, occurrence, analytical strategy, toxicity, detoxification

## Abstract

Synthesized by the secondary metabolic pathway in *Aspergilli*, aflatoxins (AFs) cause economic and health issues and are culpable for serious harmful health and economic matters affecting consumers and global farmers. Consequently, the detection and quantification of AFs in foods/feeds are paramount from food safety and security angles. Nowadays, incessant attempts to develop sensitive and rapid approaches for AFs identification and quantification have been investigated, worldwide regulations have been established, and the safety of degrading enzymes and reaction products formed in the AF degradation process has been explored. Here, occurrences in feed commodities, innovative methods advanced for AFs detection, regulations, preventive strategies, biological detoxification, removal, and degradation methods were deeply reviewed and presented. This paper showed a state-of-the-art and comprehensive review of the recent progress on AF contamination in feed matrices with the intention of inspiring interests in both academia and industry.

## 1. Introduction

The link between global health and food and feed safety has been well recognized and reinforced by the One Health approach. In this system, the health of animals, humans, and ecosystems may be considered the vertices of an equilateral triangle, interdependent and inextricably linked. The tools of this harmonic balance are prevention, detection, preparedness, response, and management [1]. In this context, the reduction and prevention of the entry of hazardous substances in the early stages of the production chain, such as the primary production of feed and animal feeding, as well as the impact of animal feed on food safety, were identified as a public health priority [2,3].

Hazards, including mycotoxins, pesticides, polycyclic aromatic hydrocarbons, toxic trace elements, and microorganisms, are constantly detected by competent authorities in feed materials. The direct exposure of animals and the indirect exposure of consumers to animal-origin foods represent a health, economic, and social risk [4]. A brief analysis of the notifications reported on the European Rapid Alert System for Food and Feed (RASFF) confirms these concerns. In fact, about 1000 notifications of contamination of feed were reported. Among them, fungi and mycotoxins are the main hazard categories, with the latter confirmed as the most serious. About 80% of mycotoxin notifications reported in the portal concern the presence of aflatoxins (AFs) in nuts, grains, seeds, and other raw materials for feed.

These secondary metabolites are produced mainly by *Aspergillus* spp. fungi in certain environmental conditions, while other congeners are produced in vivo as a consequence of the ingestion of contaminated food/feed [5]. The most common *Aspergillus* species capable of producing AFs are *A. flavus* and *A. parasiticus* ubiquitous in warmer latitudes, while other species are detected in specific areas: *A. nomius* (USA and Thailand), *A. pseudotamarii* (Japan), *A. bombycis* (Japan and Indonesia), *A. ochraceoroseus* (Africa), and *A. australis* (Southern hemisphere) [6].

**Figure 1:**
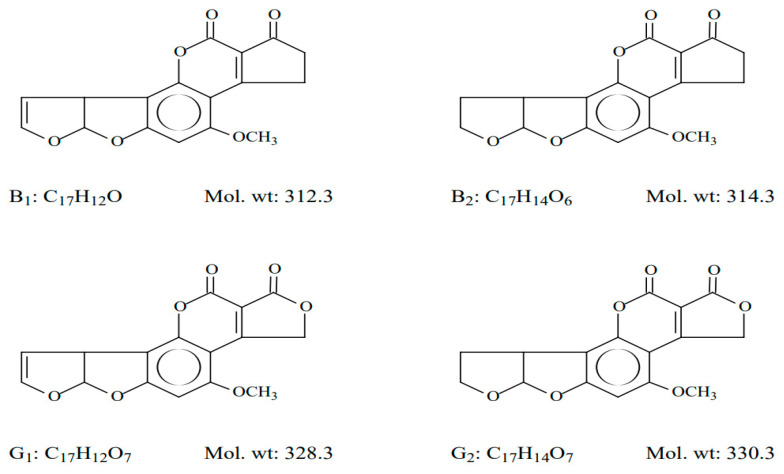
Chemical structure of most common naturally occurring AFs in feed matrices (adapted from [6]).

Characterized by a cyclopenta-difuro-chromene skeleton, which confers them high stability (melting point > 200 °C), AFs are considered the most hazardous chemicals due to their marked acute and chronic toxicological properties [7,8,9]. The most relevant compounds detected in feed matrices, AFs of B series, B1 and B2 (intensely fluorescent in ultraviolet light, emitting blue), and AFs of G series, G1 and G2 (intensely fluorescent in ultraviolet light, emitting green), are shown in Figure 1.

Consequently, the purpose of this review is to give a comprehensive overview of the occurrence, properties, regulation, and health implications of AFs in feed commodities. The most innovative analytical procedures for their determination and the mechanisms governing mycotoxin biosynthesis and regulatory elements are also presented. This work places a special emphasis on the strategies for detoxification by biological, chemical, and physical methods. In particular, attention for these AFs removal and biodegradation using microorganisms is renewed not only for their safety, specificity, efficiency, and low cost, but especially because they do not alter the organoleptic properties of food/feed and, at the same time, are environmentally friendly and meet the actions proposed by the United Nations General Assembly resolution Agenda 2030 for Sustainable Development [10].

## 2. Ecology of *Aspergillus* Section Flavi

The AFs-producing fungi, mainly from the *Aspergillus* section Flavi, are abundant in nature and readily infect various food commodities, thereby affecting humans’ health [11,12,13]. *Aspergillus* section Flavi includes thirty-three species, and most of them are natural AF producers [14]. Members of this section can occur in the soil as sclerotia, conidia, or mycelia in plant tissue. In addition, the sclerotia of *A. flavus* and *A. parasiticus* can be produced naturally in crops by an asexual or sexual phase and, during harvest, are detached onto the soil [15,16]. Sclerotia can persist in harsh environmental conditions in the field and germinate into mycelia, followed by the formation of conidiophores and conidia [17]. In natural environments containing fertilization in soil and crops, *A. flavus* field populations have the potential for sexual reproduction. In this case, sclerotia played a role in sexual reproduction by containing many ascospore-bearing fruiting bodies, termed cleistothecia, following fertilization by a sexually compatible strain [18]. The exchange of genetic materials during sexual recombination results in high genetic diversity in the *A. flavus* population. Thus, mycotoxin production and vegetative compatibility groups in *A. flavus* are more diverse as compared to other species in section Flavi [13].

Temperature and aw have a noteworthy consequence on the growth of *Aspergillus* section Flavi and the subsequent AF production [19,20,21]. The minimum aw for *A. flavus* growth was reported to be at 0.91 a_w_ at 25 and 37 °C in sorghums [22], while the minimum a_w_ in paddy was predicted between 0.83 and 0.85 [23]. A lower growth rate was observed at a_w_ < 0.85 or temperature < 20 °C, while better growth was observed at a higher aw and around 28–40 °C [13].

## 3. Toxicity of AFs

AFs pose the most significant food safety concern since they are disseminated in foods and feeds and are highly toxic [9].

### 3.1. Genotoxic Effects

AFB1 exhibits mutagenicity in *Salmonella typhimurium* strains TA98 and TA100, with its mutagenic impact boosted around 1000-fold when S9 is present [6], highlighting the significance of the bioactivation system. When exposed to AFB1, both rat and human cells undergo various genetic changes, including gene mutations, abnormalities in chromosomes, the formation of micronuclei, sister chromatid exchanges, and unscheduled DNA synthesis [24]. In terms of in vitro genotoxicity, recent research has delved into the role of different CYP enzymes in the process of AFB1 bioactivation. Following exposure to AFB1, the formation of DNA adducts and an increase in recombination levels were observed in the DNA repair-deficient *Saccharomyces cerevisiae* rad4 rad51 strain, but only when it expressed the human CYP3A4 enzyme [25]. In another study, a comparison of the genotoxic potential of various AFs on cell lines revealed the following order of potency: AFB1 > AFG1 > aflatoxicol > AFM1 [26]. AFB1 caused the most genotoxicity. An AG1521 fibroblast study revealed mutations in TP53 due to AFB1 exposure, occurring mainly in specific hotspots [27,28]. Overall, recent research has reaffirmed AFB1 genotoxicity and highlighted the role of CYP3A4 in it.

In a study by Corcuera et al. [29], they observed a notable increase in micronuclei in the bone marrow and single-strand breaks (SSBs) in the liver of male Fischer rats after administering a single oral dose of AFB1 at a dosage of 0.25 mg/kg bw. Specifically, SSBs notably increased after being cleaved by the bacterial enzyme formamidopyrimidine-DNA glycosylase (Fpg), suggesting AFB1 causes oxidative DNA damage. The authors propose that these sites sensitive to Fpg might result from Fpg recognizing and cutting AFB1-FAPY lesions. Another series of studies focused on the effects of AFB1 exposure during pregnancy and early life, examining its impact on mouse fetuses. Pregnant gpt delta B6C3F1 mice were given AFB1 either through intraperitoneal (i.p.) injection or oral administration on gestation day 14. Levels of AFB1-N7-gua and AFB1-FAPY were measured in liver DNA six hours after dosing. Another group of offspring was analyzed for mutations in the gpt gene at 3 and 10 weeks of age. Comparable effects on DNA adduct levels were observed after oral administration, albeit with total adduct formation being approximately 2.5 times lower. The relative risk of gpt mutations in fetuses and adult livers resulting from AFB1-DNA adducts indicates a greater mutational impact on fetuses [30]. The influence of pregnancy on AFB1 exposure was also examined in C57BL/6 J mice, revealing higher AFB1-N7-gua adducts in the livers of pregnant mice compared to non-pregnant mice, along with increased expression of CYP1A2 and the mouse equivalent of CYP3A4 [31]. Male gpt delta B6C3F1 mice treated with AFB1 during infancy exhibited a similar level of DNA damage and mutations in the livers of both genders. In another study, AFB1-induced mutations were investigated in a different gene, showing the dominance of G-to-T transversions [32]. For Big Blue B6C3F transgenic mice, those treated with AFB1 at 4, 7, and 10 days after birth exhibited much higher mutation frequency at the *cII* gene in the liver, especially G-to-T transversions. However, AFB1-treated adult mice did not show an increase in mutation frequency, but G-to-T transversions significantly rose in molecular analysis. These results align with a previous report on neonatal mice’s lower GST levels, linked to increased AFB1-DNA adduct formation [33]. In summary, pregnancy enhances AFB1 genotoxicity for mothers due to elevated CYP1A2 and CYP3A4 levels. Utero exposure showed lower DNA adduct frequency in fetuses than in mothers, but mutation frequency was significantly higher, indicating a greater mutational impact.

### 3.2. Mutagenic Impact

Numerous studies have explored the mutagenic impact of chronic infections like HBV and HCV, along with exposure to AFB1 via diet, on the development of liver cancer. A key mutation site in the TP53 gene is codon 249, prone to AFB1-induced mutations, primarily a G-to-T change (AGG to AGT, R249S). In hepatocellular carcinoma (HCC) cell lines, the R249S variant demonstrated a capacity to attach to p53 response elements and trigger the activation of p53 target genes [34]. The presence of high R249S levels is intricately linked to HBV-related HCC, while lower to medium levels appear in asymptomatic individuals exposed to AFB1 [35]. Recent research has delved into the broader mutational patterns caused by AFB1 exposure [36,37]. Genomic sequencing of 4915 HCC cases in the high-AFB1-risk region of Qidong [38] was compared with 1072 HCCs from the general population with no known AF exposure. The prevailing mutational pattern involved increased G-to-T transversions, particularly in the GGC sequence motif and mainly on the non-transcribed strand. Frequently mutated genes included TP53, TERT, AXIN1, CTNNB1, and ADGRB1 [37]. The study estimated that AFs-associated HCCs accounted for up to 9.8% in China, 3.5% in the United States, 1.7% in France, and 0.4% in Japan. Another study used whole genome sequencing to analyze mutation patterns in human cell lines, mouse liver tumors, and mice carrying an HBV surface antigen gene. The mutation patterns were quite consistent across these systems and aligned with HCC mutations seen in high AFs exposure areas, validating mutational patterns as indicators of AFs exposure. This study specifically focused on HCC samples with somatic TP53 R249S mutations [36]. Through comprehensive genome-wide mutational signature analysis and comparisons, the authors estimated AFB1-related HCC proportions to be 0.7% in North America, 1% in Japan, and 16% in Hong Kong [36]. Therefore, whole genome sequencing assessment of mutational patterns appears to be a valuable tool for identifying AFB1 exposure in Western countries.

In terms of long-term toxicity (including carcinogenicity), AFB1 possesses clear carcinogenic properties across various animal species. The main tumors observed in rodents were primarily liver-based, particularly hepatocellular carcinoma (HCC), but tumors also emerged in the lung, kidney, and colon. The male Fischer rat was identified as the most responsive species [38]. Furthermore, it is worth noting that AFB1 and AFG1 exhibit similar levels of carcinogenicity. This conclusion is based on the fact that AFB1 has a greater capacity to induce liver tumors compared to AFG1, which is more likely to induce kidney tumors. In a study by Butler et al. [39], it was found that AFG1 caused fewer liver tumors than AFB1 but led to a higher incidence of kidney tumors in male rats. AFB2, on the other hand, did not trigger liver or kidney tumors but did result in unspecified neoplasms in some animals. No risk assessment for AFM1 was reported [40]. There is a shortage of long-term toxicity studies in rodents. Halver [41] initially suggested using rainbow trout as a test subject for cancer research. In his research, he investigated 13 potential carcinogens, including AFB1, and observed liver tumors in all trout that were fed a diet containing the carcinogen for 6–9 months. Additionally, Oregon State University utilized rainbow trout as a liver cancer model to evaluate various potential carcinogens, including AFB1. The research demonstrated that rainbow trout exhibited a consistent response to AFB1, showing a linear dose-response relationship across a wide range of concentrations [42,43].

### 3.3. Developmental and Reproductive Toxicity and Immunomodulatory Effects

In terms of metabolomics, zebrafish embryos exposed to AFB1 demonstrated toxicity, with more pronounced effects at later stages. AFB1 exposure altered various metabolites associated with amino acids, carbohydrates, neurotransmitters, fatty acids, cholesterol, and GSH levels [44]. AFB1 exposure affected brain development in rats (NOAEL: 7.1–13.6 μg/kg bw per day), caused early delivery and low birth weight in mice (NOAEL: 50 μg/kg), and impacted spermatogenesis and folliculogenesis at 4 μg/kg bw (LOAEL), indicating reproductive and developmental effects at low doses with potential short-term exposure [45,46,47,48,49,50,51,52].

AF’s immunomodulatory effects have been extensively studied in vitro and in vivo, revealing their potential to harm the immune system across various animal species. AFB1 exposure demonstrated an increased susceptibility to viral infections, with macrophage polarization from M1 to M2 and subsequent immune response changes [53,54]. According to Meissonnier et al. [55], the immunosuppressive effects of AFs led to heightened vulnerability to microbial infections and reduced vaccine efficacy. In human monocyte-derived dendritic cells exposed to AFB1 in vitro at 10 ng/mL, impaired phagocytic capacity and gene expression changes associated with metabolic pathways, immune response, and cell adhesion were observed. Similarly, human microglia cells exposed in vitro to a low AFB1 concentration (20 ng/mL) showed increased proinflammatory molecules’ mRNA expression and protein secretion of IFN-γ and GM-CSF [56,57]. The NOAELs for such effects in rodents were generally around 30 µg/kg bw [58,59].

### 3.4. Gut Microbiota

Wang et al. [60] conducted a study examining the impact of AFB1 on the intestinal microbiota of Fischer F344 rats. These rats were exposed to AFB1 through oral gavage for a period of 4 weeks, with varying doses. Their results revealed that AFB1 exposure led to a dose-dependent alteration in the gut microbiota, characterized by a reduction in microbial diversity and a specific depletion of lactic acid bacteria. Zhou et al. [61] involved the same rat strain and exposure method, and it was found that up to 4 weeks of AFB1 exposure led to changes in gut-dependent metabolism, including decreased levels of short-chain fatty acids. Yang et al. [62] exposed Kunming mice to AFB1 for 2 months and observed that all doses decreased the diversity of the gut microbiota. Higher doses led to increases in both beneficial and harmful bacteria. Similarly, He et al. [63] discovered that AFB1 exposure in Kunming mice increased bacterial populations and raised certain intestinal enzyme activities. Subsequently, AFB1 exposure at varying doses can consistently disrupt microbial communities, generally by decreasing their diversity. No studies exploring AFG1, AFG2, AFB2, or AFM1 about this aspect were found.

### 3.5. Liver Damage

AFs primarily target the liver and are metabolized into highly reactive compounds that bind to cellular components, leading to DNA damage and disruption of cellular functions. AFs may cause mutational abnormalities and reproduction disorders [64]. AFs can pass into milk, eggs, and meat, presenting a direct threat to human consumers and potentially leading to chronic health issues.

Liver triglycerides and phospholipids, as well as certain genes linked to lipid and lipoprotein metabolism, increased. Hussain et al. [65] found that AFB1 administered through gavage (0.5 or 1.0 mg/kg bw per day) for up to 40 days in adult male rats led to decreased food intake and body weight, along with liver toxicity and kidney damage. The liver showed fatty changes, cell death, and enlarged cells and nuclei. Kidney damage included tubular necrosis, increased serum ALT and creatinine levels, and decreased levels of various proteins and lipids. Limited knowledge exists about AFB1’s potential impact on the endocrine or neuroendocrine systems. Moreover, an investigation involving male Wistar rats [66] subjected to AFB1 exposure via oral administration twice a week for a duration of 5 weeks revealed a reduction in weight gain. This decrease in body weight may be associated with a lowered expression of hypothalamic neuropeptides, suggesting that AFB1 could disrupt the hypothalamus regulation of appetite-controlling neuropeptides, resulting in decreased body weight. Another study by Qian et al. [67] assessed Fischer rats exposed to repeated AFB1 doses through oral gavage over a 5-week period, with weekly monitoring. The decline in body weight became noticeable in the second week. AFB1, across all tested doses, exhibited various detrimental effects on the rodents, including hindrance of normal growth and damage to the liver and kidneys. It is worth noting that there were no identified studies focusing on short-term toxicity caused by AFG1, AFG2, AFB2, or AFM1.

In more detail, the toxicity mechanism involves the creation of chemical bonds known as adducts with DNA, RNA, and proteins. The formation of these adducts interferes with the normal functioning of cells, especially in the case of DNA adducts, which can ultimately result in a loss of control over cell growth and division [68]. Furthermore, this process also leads to the oxidation of lipids and DNA damage [69]. Once ingested, AF undergoes metabolic transformation in the liver by a group of enzymes known as cytochrome P450, resulting in their conversion to their respective epoxides. It is important to note that AFB2 and AFG2 cannot form 8,9-epoxide. The AFB1 8,9-epoxide can chemically react with DNA to form these adducts, and it is believed to be responsible for the biological effects of this toxin. Research has shown that the consumption of AFB1 orally leads to liver damage and disruption of the regulation of genes associated with lipid and lipoprotein processing [70].

**Figure 2:**
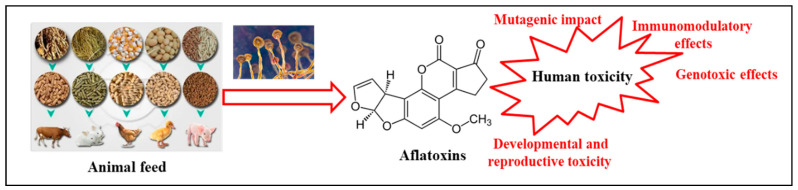
General toxicity of aflatoxins.

The toxicity of AFs in animal feeds is a complex problem that has wide-ranging consequences for human health (Figure 2).

## 4. AFs Occurrence in Food/Feed Stuff

The distribution of AFs in foodstuffs and feeds is not homogeneous, so a high contamination hotspot may occur. The most common cereals used in livestock feeds are maize and wheat [71]. Cereals contain important lipids, carbs, proteins, minerals, and vitamins essential to animal nutrition [72]. Pseudo-cereals have also been used in animal feed in recent years, as they bring significant benefits to the health of various animal species. Quinoa is the most widely used pseudocereal as an ingredient in animal feed, while amaranth is increasingly used in animal nutrition [73]. A good source of protein can also be taken by many animals, such as ruminants and dairy cattle, from raw cottonseed, which is also used as animal feed [74].

Feed contamination can occur before and after harvest [75]. Mycotoxin-contaminated animal feed can occur worldwide, and this could be due to international trade [76]. In the report recently published by EFSA in 2020 [5], it was highlighted that the monitoring of AFs should be continuous since climate change may increase their occurrence. This scientific opinion concerned results drawn from more than 25 European countries. According to the results of this report, it appears that the change in climate conditions will increase the presence of AFs from low to moderate in food in Europe, while in countries such as France, Italy, and Romania, which are major maize-producing countries, there will be a greater increase [5].

In 2022, RASFF reported that mycotoxins were the third most reported risk category, after pesticide residues and pathogenic microorganisms [77]. Specifically, 485 notifications were recorded, showing an increase of 10.5% compared to 2021. Among these notifications, no AF was related to feed, and the 234 registered feed notifications in 2022 in feedstuffs were related to other risks. Based on data retrieved from the food risk prediction platform FOODAKAI [78], the presence of AFs in feed additives and feed materials as described by the EU RASFF notifications from 4 January 2016 and 2 March 2022 involved a very small percentage of 0.43%. In fact, this is the fifth position behind nuts, nut products, and seeds (74.28%), fruits and vegetables (13.26%), herbs and spices (8%), and cereals and bakery products (3.27%). Cows fed with AFB1-contaminated feed pose a significant threat to public health as the milk produced contains the mycotoxin AFM1, which is the hydroxylated metabolite of AFB1 at a rate of 0.35–6.2% [79,80]. Environmental factors such as temperature and humidity are among the most critical factors that affect the increased contamination of *Aspergillus* mycotoxins [81,82]. From the beginning of the 21st century until today, it is believed that climate change has contributed to the increased presence of *Aspergillus* mycotoxins in southern Europe [81]. In Northern Europe, a steady increase in cases of aflatoxin contamination was also observed. AFB1 in maize and AFM1 in milk have been the two most frequently detected aflatoxins in the past decades, while many European countries have reported an increased incidence of aflatoxin [83,84].

In the previous decade, hot summers without rainfall caused significant contamination of maize with AFB1, which in turn led to contaminated quantities of milk and dairy products with AFM1 in Serbia in the years 2012, 2013, 2015, 2017, and 2021 [85,86]. The impact of climate change also resulted in the occurrence of AFs in maize in the 2018–2020 crop seasons in Serbia and Croatia at rates of 10% and 20%, respectively [87].

In Ethiopia, oilseed cake is largely exploited for feed, which is extremely susceptible to AF adulteration [88]. This research showed that all samples were infected with AFB1 (7–419 µg/kg). Eighty-eight percent of samples infected by AFB1 surpassed the EU AFB1 upper limit (5 µg/kg) [89]. Hashemi [90] evaluated the occurrence of AFB1 in various Iranian feed samples. These authors performed the quantitative analysis of AFB1 by competitive ELISA. The overall AFB1 level was 4.12 μg/kg, and 11% of samples showed AFB1 ≥ 5 μg/kg [91,92]. By HPLC-FLD, Bahrami et al. [93] determined the AFs in the feedstuffs of dairy cows. AFs were identified at levels between 41 and 82% of feed samples, and several ones presented concentrations superior to the EU limit. AFB1 in feed ingredients and complete pig feeds from different regions of China from 2016–2017 was determined by Ma et al. [94]. AFB1 demonstrated a moderate occurrence in the examined samples with an interval of 74–100% during the two years. A comparatively lower mean level of AFB1 (1.6–10.0 µg/kg) was restrained in the existing study compared to previously reported levels (0.4–627 µg/kg) in China during 2012–2015 [95].

According to a summarized report, the series values of AFB1 in feed materials and feedstuffs were reported to be 8 µg/kg, 2–3 µg/kg, 0–3 µg/kg, 8–90 µg/kg, 1 µg/kg, and 42 µg/kg in North America, Central South America, Europe, Asia, Oceania, and Africa, respectively [89]. A similar array value of AFB1 in feedstuffs in the current study to the value from Asia was reported (0–67.6 µg/kg), which was the greatest compared with other zones [89]. Changes in seasonal and local weather situations through critical plant growth stages might be the critical factors for modifications in the occurrence of AFB1 between geographical areas. 2.1% of feed ingredient samples (>20.0 µg/kg) and 14.6% of complete pig feed samples (>5.0 µg/kg) exceeded the EU regulation [95]. Kosicki et al. [96] evaluated mycotoxins in Polish raw materials and animal nutrients. Only one sample showed a level equal to the recommended LOQ. Segvic Klaric et al. [97] analyzed twelve samples of maize and identified AFs at concentrations of 2.7–4.5 μg/kg. Martins et al. [98] explored the AFB1 occurrence in thirteen silage samples. Fifteen percent of samples are infected by AFs (6–10 mg/kg). Sahin et al. [99] detected AFs in dairy cow feed (76 samples) using HPLC-FLD. AFs were present in 26.3% of feed samples, and 2 feed samples exceeded the maximum limit (ML) of 5 µg/kg for AFB1 as established by the EU. Agbetiameh et al. [100] examined the prevalence of AF contamination in maize and groundnut in Ghana. For AFs analysis, homogenates were directly spotted on thin layer chromatography (TLC) plates, developed with diethyl ETH-MeOH-water (96:3:1), and visualized under ultraviolet light at 365 nm. AFs were quantified directly on TLC plates with a scanning densitometer and quantification software. 15% of maize and 11% of groundnut samples exceeded the AFs threshold limits set by the Ghana Standards Authority of 15 and 20 ppb, respectively [101]. Abdallah et al. [102] investigated the contamination by AFs in 61 Egyptian samples of maize and 17 commercial animal feed samples. Sample cleanup was carried out by multi-mycotoxin IAC (AOZ IAC) columns. Detection of the target toxins was achieved by mycotoxin purification and an HPLC–FLD system with on-line post-column photochemical derivatization. AFB1 was detected in maize and feed; however, only one maize sample exceeded the maximum allowable level set by the Egyptian authorities. The presence of AFB1 in maize from Egypt at levels higher than the national and international limits was confirmed. These results were in accordance with previously reported levels in maize (19.2 µg/kg) [103]. The AFB1 level in feed was lower than those reported in feedstuffs from different regions of Egypt, where up to 400 µg/kg were detected [104] and from Assiut-Egypt, where 60 µg/kg were detected [63]. On the other hand, AFB2 was observed in six maize samples and in one feed sample, with a maximum value of 0.5 µg/kg. AFG1 and AFG2 were under the limits of detection. The occurrence of *Aspergillus* spp. (75%), *Fusarium* spp. (11%), *Penicillium* spp. (8%), and *Trichoderma* spp. (6%) in 150 maize samples collected from Southern Nations Nationalities and Peoples (SNNP) regions [105] was also reported. AFs were detected in the range of 20–91.4 µg/kg in 100 samples, showing a higher incidence of AFs-producing fungi and implying the presence of a high level of AFs beyond the EU maximum limit in maize samples used as an ingredient in foodstuffs [106]. Multi-mycotoxins and fungal analyses carried out in 100 maize samples from south and southwestern Ethiopia [107] revealed that these samples were contaminated by *Penicillium* spp. (80%), *Aspergillus* spp. (75%), and *Fusarium* spp. (60%). Besides, AFs were detected in 2–8% of the total samples.

The impacts of climate change on Aspergillus species emergence and AF production are uncertain. Furthermore, if climate change remains in its current situation, AF contamination risks in fields will increase or decrease depending on the region under consideration [108]. Recurrently, species of *Aspergillus* most occur in regions with tropical and subtropical climates, most commonly found in latitudes between 25 and 35 °C, and therefore the AFS occurrence was most significant [109]. The a_w_ of 0.99 is the optimal environmental condition for the production of aflatoxin [81]. For example, Atukwase et al. [110] showed that high-altitude regions were linked with the highest total AF contamination in crops, while crops cultivated at mid- and low altitudes had less mycotoxin contamination. Additionally, Fapohunda and Adewunmi [111] reported that new and rare species of *Aspergillus* have been reported frequently from tropical and subtropical soils. It should be noted that in developing countries, the presence of AFs is a significant problem as economic development is limited and quality controls of agricultural products are absent [112]. Entomological infestations can also contribute to the occurrence of aflatoxins as the incidence of toxic fungi increases [113].

Table 1 summarizes some recent studies that have been conducted for the detection of AFs in animal feed in some countries, such as China, Pakistan, and the Philippines (Asia), Tunisia, Ghana, Nigeria, and Ethiopia (Africa), Mexico (North America), and Spain (Europe).

## 5. Chromatographic Technologies for AF Detection in Feeds

For quantitative analysis of mycotoxin in cereal-based food samples, chromatographic techniques showed a group of techniques most commonly used, which are highly selective, sensitive, and accurate [123]. Chromatography tools have been utilized to assess AFs in feed. Among them, HPLC-FLD is extensively used since it has been applied to many official methods [114]. Also, HPLC-FLD costs and maintenance are reasonable for most labs. For instance, Mushtaq et al. [124] showed the presence of AFs using HPLC-FLD in selected cereal-based processed products and reported an LOQ of 0.02 ng/g. Muñoz-Solano and González-Peñas [122] developed an LC-FLD-based approach proper for AFs presented in animal feed. LODs were 2 (for AFB1 and AFG1) and 0.64µg/kg (for AFB2 and AFG2), while the recovery % was 73 and 88%. With a view to purifying the animal feed extracts prior to analyzing AFs by LC-FLD, Khayoon et al. [91] employed a multifunctional column (MFC). As a mobile phase: MeCN/MeOH/water (8:27:65, *v*/*v*/*v*), in isocratic conditions, these authors separated four AFs (AFB1, AFB2, AG1, and AG2) within 30 min. To assess AFB1, AFB2, AG1, and AG2 in peanuts, Blesa et al. [92] developed matrix solid-phase dispersion (MSPD) using C18 as the dispersive adsorbent and MeCN as the elution solvent. LOQs varied between 125 and 2.5 ng/g for all AFs through LC–FLD. In addition, LC-MS-ESI was applied to confirm AFs [92]. A method focused on AF analysis without involving any post-column derivatization was established by Oulkar et al. [125]. In order to provide satisfactory extraction efficiency and improved precision in comparison to dry homogenization, a slurry of the sample was required to be developed. The method workflow involved the addition of MeOH-water (80:20) to a portion of the slurry, its extraction, followed by IAC cleanup through AF-specific cartridges, and finally, its analysis by UHPLC-FLD. The requirement of any post-column derivatization could be eluded by the use of a large volume flow cell (13 μL) in the instrumentation method employed by NRL-NRCG. Millets, rice, and corn have been evaluated by this standardized and validated method. In this study, the LOQ ranged between 0.01 and 0.025 ng/g in peanuts. The retention times of AFs were less than 4 min. To detect AFs in animal feeds, Kumar et al. [94] employed FLD UHPLC and confirmed that for each AF, the LOQ = 0.5 ng/g and recoveries were above 75%. In some studies, pre- and post-column derivatizations are used to improve sensitivity. In this line, Hu et al. [95] analyzed AFs in high-pigment samples and involved MSPD and HPLC-FLD with KOBRA cells. LODs are between 0.1 and 0.25 ng/g. Microwave-assisted extraction (MAE) has been evaluated for AF analysis. Chen and Zhang [40] used the MAE [MeCN at 80 °C/15 min] as a pretreatment tool for AFs analysis in grains of a total of thirty-six samples collected from Chinese markets. Samples were cleaned up by SPE, followed by TFA, and AFs were identified by HPLC. The dominant mycotoxin was AFB1 at 3.41–42 μg/kg. The cost and maintenance of the instruments for MAE were significantly higher than conventional lab glassware used for liquid extraction.

Atmaca et al. [126] evaluated the AF in maize grain. LOQs for Kobra cell derivatization ranged between 0.007 and 0.063 ng/g. For the PHRED derivatization, the LODs were 0.08, 0.02, 0.09, and 0.02 ng/g for AFB1, AFB2, AFG1, and AFG2, respectively, and the LOQs were 0.17, 0.04, 0.22, and 0.04 ng/g for AFB1, AFB2, AFG1, and AFG2, respectively [126]. Furthermore, the LODs for some cereal samples are reported to be 0.5 ng/g (AFB1 and AFG1) and 1.0 ng/g (AFB2 and AFG2) [127]. Torović [128] analyzed AFB1 in different types of Serbian flour marketed and analyzed using HPLC-FLD. In corn flours, the % of positive samples varied significantly over the years, and AFB1 varied from 7.1–80.0%. The highest recorded levels were 8.80 μg/kg of AFB1 (corn); in addition, the overall mean contamination levels of corn flours were equal to 0.53 μg/kg. Table 2 summarizes an overview of emerging detection chromatographic techniques used to analyze AFs in feeds.

Different management strategies have been employed for the control of these AFs worldwide. In this way, efforts to mitigate AF toxicity in feeds involved various strategies, including pre-harvest and post-harvest management practices.

Pre-harvest measures include proper crop rotation, timely harvesting, and the use of resistant crop varieties. Post-harvest interventions encompass proper storage and handling to prevent mold growth and toxin production.

Post-harvest contamination by aflatoxigenic fungi was influenced by storage conditions, which trigger serious damage and AF accumulation at levels higher than international accepted ones [142]. Changes in water activity (a_w_) were key in determining the effects that different postharvest actions will have. AFs-producing *Aspergilli* such as *A. flavus*, *A. carbonarius*, and *A. ochraceus* are competitive with other microbial food spoilers at an a_w_ below 0.8 [143]. Germination occurs over a wider range of a_w_ than that for growth, whereas AF production ranges are slimmer than the growth [142]. The optimal conditions for AF production by *A. flavus* and *A. parasiticus* are 33 °C and a_w_ = 0.99, while those for growth are 35 °C and a_w_ = 0.95 [143]. The influence of environmental factors on the growth of *A. flavus*, *A. parasiticus*, and *Aspergillus oryzae* was comparable, with a_w_ = 0.82 at 25 °C and a_w_ = 0.81 at 30 and 37 °C [143]. Current management practices can reduce the incidence of post-harvest AF contamination, including drying, storage, and processing. Generally, drying can inhibit the growth of spoilage microorganisms and stop the occurrence of browning and other moisture-driven deterioration reactions. However, poor drying can result in *Aspergilli* colonization and potential AF contamination. For instance, harvesting of maize is often carried out at moisture contents that are >14–15%, which requires drying to reduce the available water to <0.70 a_w_ (=14%), which is safe for storage [143]. To prevent the initiation of *Aspergilli* species, drying to a_w_ = 0.70 is crucial [143].

Stringent control methods, thorough surveillance, and successful measures to address the issue are crucial for protecting livestock, ensuring food safety, and reducing the economic and health hazards linked to AF contamination in animal feed.

## 6. Prevention and Detoxification of AFs in Different Feed Matrices

Feed safety is directly linked to animal and human health. It requires an integrated and holistic approach from production to consumption. This comes from the correct training and information of crop and livestock workers and managers. Good agricultural, farming, and hygiene practices are essential since the AFs may be introduced at different stages of the feed chain—from on-farm production to post-production, transport, storage, mixing, and distribution [131].

### 6.1. Prevention of AFs in Feed Matrices

At the field level, the prevention of plant contamination by AFs includes crop rotations, proper tillage, correct fertilizer campaigns, and control of humidity, temperature, and vectors [144]. Another important pre-harvest intervention is the utilization of transgenic crops. In particular, Bt corn, a genetically modified organism, was developed in 1990 with the primary purpose of protecting crops against insects through the insertion of genes from *Bacillus thuringiensis*, codifying for delta endotoxins and other proteins [145]. Several studies showed that the reduction of insect damage on corn plants was directly correlated with a lower fungus infection and the occurrence of mycotoxins in Bt corn [146]. The evidence that AFs levels were significantly lower in biotech corn was confirmed by recent studies [147]. Other advanced biotech technologies and transgenic crops were recently developed; as an example, the antifungal activity of transgenic maize expressing the α-amylase inhibitor from the bean *Lablab purpureus* was investigated, and a great reduction of the concentration of AFs between 62 and 88% was achieved [148].

Genetic manipulations, such as the usage of CRISPR (clustered regularly interspaced short palindromic repeats) technologies, are under investigation, both to produce resistant mycotoxin-infection corn and to introduce competitive fungi species that are not capable of producing mycotoxins [149,150]. Many of these atoxigenic *Aspergillus* spp. strains were evaluated as biocontrol agents in several countries. Probably one of the most known is the non-aflatoxigenic strain of *A. flavus*, MUCL54911, developed for field application as the result of several AF outbreaks in the maize for feeding grown in Italy, which heavily damaged the Italian dairy industry due to the carry-over effect [150,151].

After a temporary authorization in 2015, in 2018 and 2020, a complete dossier of activity, safety, and risk assessment of this new active substance, in accordance with European Regulation (EC) No. 1107/2009 [152], was submitted for the approval of the European Commission. Despite the promising results, data gaps on persistence, transformation, and mobility in the environment were identified by the EFSA, and the approval is still pending [153,154]. Similarly, other non-toxin-producing strains of *A. flavus* inserted in complex biocontrol products were developed and tested. As an example, the product Aflasafe^®^ was developed primarily for sub-Saharan Africa and is composed of sterile sorghum grains coated with spores of four native atoxigenic strains of *A. flavus*. Several studies showed that when applied in the field, the atoxigenic strains displaced most of the toxigenic fungi residing in the treated field, resulting in a significant decrease in aflatoxin content in those crops [152]. Other atoxigenic biocontrol products, such as *Aspergillus flavus* AF36, JS4, SI1, SXN, PA04, and PA10, were successfully used in affected crop areas of the United States (e.g., California), China, and Africa (e.g., Kenya) [155,156,157,158].

In this way, the application of atoxigenic *A. flavus* strains has the potential to become the most successful aflatoxin biological control approach. In particular, the researchers observed that most *A. flavus* belonging to the subgroup of L-strain (sclerotia diameter > 400 µm) do not produce AFs, so their soil inoculation interferes with the proliferation of toxigenic strains [159].

Furthermore, the usage of specific small interfering RNA molecules (RNA-interference gene cassettes) that downregulate and silence genes involved in the *Aspergillus* AFs biosynthetic pathway, such as the transcription factor *aflR* and the gene *aflC*, is another attractive and promising strategy to provide a sustainable solution against mycotoxin contamination [159,160,161,162].

Moreover, among these pre-harvest techniques lies the use of specific plant protection products (PPPs) that inhibit fungal growth (fungicide action). Although there are not specific PPPs registered for *Aspergilli* control, there are thousands of studies that have shown the high efficacy of several residues, such as boscalid, azoxystrobin, prothioconazole, tebuconazole, cyprodinil, and fludioxonil, in reducing *Aspergilli* growth, sporulation, and AFs production [163,164]. In recent years, these substances have been under investigation due to their human and environmental toxicity, so their usage has been discouraged and limited in many countries. Furthermore, several studies showed that *Aspergilli* fungi develop mechanisms of resistance to some classes of fungicides [165].

### 6.2. Post-Harvest Intervention Strategies

After the harvest, continuous testing of batches of raw materials, premixes, and other ingredients of feed appears mandatory. However, in the last few years, a more proactive strategy has helped the agricultural and livestock industries ensure and even restore feed safety and prevent economic losses resulting from the rejection and disruption of contaminated commodities. Many technologies have been developed to decontaminate feed commodities and detoxify AFs using physical, chemical, and biological methods that cause the inactivation, absorption, sequestration, and removal of AFs. The characteristics of the optimal treatment include high efficacy and safety, and they should not alter the nutritional values or cause deterioration of the final product.

#### 6.2.1. Physical Treatments

Among the simplest technologies that may be used to reduce AF concentrations in feed commodities are cleaning, sorting, and peeling. Although AFs are not soluble in water, there are many studies indicating that contaminated feed has a different density, size, and weight. So, apart from manual sorting by visualization, sorting by washing or sieving may be good strategies to reduce AF, especially in raw and premixing materials [166,167]. Apart from the removal of damaged nuclei, the peeling of raw materials may be used. In this context, several devices and technologies were developed based on fluorescence/laser detection. Several portable devices were tested for sorting maize kernels [168,169].

Although AFs are characterized by high chemical structure stability, it is reported that AFB1 concentration may be reduced until 80–90% heating the materials at temperatures > 200 °C. However, this approach may alter the biochemical composition of feed matrices [170,171]. Another efficient technique is the use of radiation. In particular, different studies using different sources of γ radiation (mainly ^60^Co or ^137^Cs) and a range of radiation doses from 0.5 to 60 kGy were carried out using many feed substrates. However, Di Stefano et al. [172] underlined that the maximum reduction of AFB1, AFB2, AFG1, and AFG2 in animal feeding stuff using a dose of 15 kGy is around 25%. So, this process, despite its effectiveness, may require doses that alter the nutritional composition, especially the lipidic fraction, of feed. Other studies reported a dramatic depletion (80–90%) of AFB1 contents by using a range dose of 10–25 kGy in barley, bran, and corn feed [166]. Similarly, Markov et al. irradiated maize feed, intentionally contaminated with 50 μg kg^−1^ AFB1, at 10 kGy, obtaining a reduction of more than 90% of AFB1 content [171,172]. However, in most of this research work, the modifications in the nutritional composition of matrices were not assessed. Other studies reported the use of UV light since AFs are degraded under these conditions. According to Faraji et al., a treatment of 30 min and 20 cm distance with a UV light source reduced *A. flavus* and *A. parasiticus* contamination in rice and helped minimize AFs contamination [173].

#### 6.2.2. Chemical Agents

The use of small molecules mainly with acidic, basic, or oxidative properties is the most ancient method to treat contaminated feed commodities with mycotoxins. One of the most recognized treatments is ammonia. Treatments with ammonia in the gaseous phase or in solutions at various concentrations, as well as the use of ammonia-releasers, demonstrated their effectiveness in many studies and/or in combination with other physical and chemical treatments. In some legislation, the treatment is authorized on feed and some seeds/spices, as well as the subsequent use of ethylene oxide in the gaseous phase or formaldehyde. In recent years, this time of treatment has been less used due to the production of harmful reaction sub-products [64].

On the contrary, more recently, the usage of ozone treatments has been increasing due to its antimicrobial properties, the high oxidation power of several mycotoxins, including AFs, and the fact that it does not produce residues [174,175]. Although some authors reported that AFB1 and AFG1 were sensitive to ozone and easily degraded with 1.1 mg/L of ozone within 5 min at room temperature, other studies indicated that application of ozone in the range of 6–90 mg/L for a variable time, from 20 min to 96 h, reduced AFs in a quantitative manner (>90%) [176].

Another completely different approach to reducing AFs in feed is the addition of absorbents, which can take place at all stages of the harvesting and handling of feed. The contaminant is bound by substances with a sequestering action and then removed from the plant or during the feeding phase [177,178]. Table 3 shows the various organic and inorganic substances used for this purpose. Preparations and combinations of active substances are on the market and approved as feed additives. In fact, EC No. 1831/2003 [179] has established the possible usage of “substances for reduction of the contamination of feed by mycotoxins: substances that can suppress or reduce the absorption, promote the excretion of mycotoxins, or modify their mode of action”. Studies in mammals and fish have shown that chlorophyll can inhibit the formation of carcinogens through the combination of AFB1, reduce tissue bioavailability, and finally reduce the incidence of tumors. However, prolonged intake of these materials may also result in malabsorption or weight reduction in animals. For some clays administered together with feed, there are very in-depth safety and toxicity studies (e.g., NovaSil), which have shown that the benefits of the treatment undoubtedly outweigh the mild side effects encountered [64].

Among chemical treatments is the use of active substances extracted from plants/animals, which permit both to reduce fungal growth and to inactivate/sequester AFs. Many studies about the utilization of polyphenols, glucosinolates, carotenes, proanthocyanidins, essential oils, and extracts were carried out [180,181]. However, complete efficacy, safety, and toxicological studies of the application of these substances as feed additives are lacking and should be implemented.

#### 6.2.3. Biological Methods

Microbial degradation was considered an attractive method due to its specificity, efficiency, environmental friendliness, protection of the quality and flavor of food, and feasibility of the processes when applied in industries. The most common mechanism is the inoculation of strains of bacteria, yeasts, and fungi that bind or degrade AFs. Microbial enzymes and proteins bind or degrade the mycotoxin, or, as an alternative mode of action, cell wall components sequester it [182]. Several species of *Bacillus* and *Lactobacillus* possess enzymes capable of degrading AFB1 with an efficacy of up to 90%. *Bacillus subtilis* ANSB060 administered with a moldy peanut meal naturally contaminated with AFs to broilers has shown a protective effect and a marked reduction of AFs in chicken livers. Similar results were reported in other studies [183,184]. Thanks to the production of lactic acid and other small molecules, lactic acid bacteria (LAB) have proved to be further effective both in blocking fungal growth and in the degradation of various classes of mycotoxins; therefore, their use could be particularly useful in cases of cross-contamination [185]. Ma et al. [186] tested the AFB1-binding capacity of 10 bacteria in contaminated corn silage, including three *Lactobacillus plantarum* strains, two *Pediococcus acidilactici* strains, and *Lactobacillus buchneri* R1102. A linear decrease in AFB1 concentration was observed in all samples within 3 days of incubation. Similarly, *L. acidophilus, L. brevis, L. casei, L. delbruekii,* and *L. plantarum* were inoculated in AFB1-contaminated maize samples at 37 °C. After 5 days, a pronounced AFB1 reduction was observed by the researchers [187]. Liu et al. achieved a biodegradation of 83.4% AFB1 in cottonseed poultry meal by inoculating 10^8^ cfu/mL *Cellulosimicrobium funkei* for 144 h at 35 °C [188]. *Saccharomyces cerevisiae* and *Rhizopus oligosporus* were used to remove AFB1 in chicken feed [189]. Zhou et al. [190] screened out and identified *Zygosaccharomyces rouxii* from fermented soy paste—one kind of traditional Chinese food—to detoxify AFB1 by aerobic solid-state fermentation in peanut meal.

Another approach that deserves further exploration is the enzymatic detoxification of AFs using enzymes extracted from microorganisms. In fact, although enzymes for the removal of AFs from feed have not yet been approved in major world legislation, this line of research should be implemented. As an example, laccases from *Trametes versicolor* and *Pleurotus pulmonarius*, manganese peroxidase from *Pleurotus ostreatus*, and oxidase from *Armillariella tabescens* have been used for the enzymatic detoxification of AFB1 [190].

A comprehensive overview of the prevention and detoxification strategies of AFs in feed matrices is shown in Table 3.

**Table 3:** Prevention and detoxification strategies of AFs.

Rationale	Advantages	Treatment	Conditions (Measures/Reagents)	Pre-Harvest	Storage	Processing/Mixing	Feeding	References
**Agricultural Prevention**								
Interventions that prevent/deplete fungi infection or inhibit AFs production.	Prevention Low cost High durability	GAPs	tillage—farming planning crop rotation fertilizers biostimulants	×				[179]
		Pesticides	azoles boscalid azoxystrobin cyprodinil fludioxonil	×	×			[185] [186]
		Transgenic crops	GMO RNA interferences CRISPR	×				[152,153,154,155]
			*Atoxigenic A*. spp strains	×				[155,156,157,158]
		Bioremediation of fields	degradation transformation	×				[170]
**Physical Methods**								
Interventions that permit to remove infected/contaminated commodities or kill/inactivate fungi and spores	Standardized protocol High efficacy, No harmful residues produced	Irradiation	gamma radiation UV lights X-rays Electrons	×	×	×		[188] [189] [183]
		Drying/Thermal treatment	microwave conventional heating		×	×		[181]
		Separation/sorting	manual sieves washing light-interaction-based devices		×	×		[179] [180]
**Chemical Methods**								
Segregation, deactivation and degradation of AFs	Low cost	Ammonia	gaseous or liquid ammonia (usually 1.5–2%) alone for long time (until 15 days) or combined with other techniques	×	×	×		[36]
		Ozonisation	ozone 1.1–90 mg/l for a variable time (5 min–96 h)	×	×	×		[186] [187]
		Alkalinization	potassium or sodium hydroxide ammonium carbonate		×	×		[189]
		Acidification	lactic acid propionic acid hydrochloric acid acetic acid citric acid phosphoric acid tartaric acid sorbic acid		×	×		[181]
		Nixtamalization	Cooking heat treatment+ alkaline solution (NaHCO_3_ and Ca(OH)_2_) + H_2_O_2_		×	×		[189] [189]
		Active substances	turmeric powder curcumin resveratrol lycopene chitosan	×	×	×	×	[190]
		Feed additives	acids antioxidants polymers sodium bisulfite		×	×	×	[116]
		Adsorbents	clays activated charcoal bentonite montmorillonite, zeolite, hydrated sodium calcium aluminosilicate, kaolin, illite	×	×	×	×	[189] [190]
**Biological Methods**								
Inoculation of microbial strains in feed substrates.	Biodegradability Biosafety effectiveness regenerability	Bacteria	*Bacillus* spp. *Lactobacillus* spp.	×	×	×		[187] [187]
		Yeast and Fungi	*Saccharomyces cerevisiae* *Rhizopus oligosporus* *Zygosaccharomyces rouxii*	×	×	×		[189] [188]
		Microorganism exudates/supernatants	*Lactobacillus* spp.	×	×	×		[190]
		Enzymes	laccase manganese peroxidase oxidase	×	×	×	×	[188]

× Applied method phase for AFs prevention/detoxification. CRISPR: clustered regularly interspaced short palindromic repeats; GAPs: Good agricultural practices; GMO: genetically modified organism; NaHCO_3_: sodium hydrogen carbonate; Ca(OH)_2_: calcium hydroxide; H_2_O_2_: hydrogen peroxide.

## 7. Conclusions and Future Perspectives

The world’s demand for raw materials regularly used in the manufacturing of feed, such as wheat, maize, rice, and soybeans, has been gradually increasing in the last few years, driven by higher demands for livestock production. This has incited to an amplified alertness of feed safety problems by virtue of feed consumption is a potential path for mycotoxins hazards to enter the human food chain. Within these hazards, AFs deserve some prominence as the most prevalent and worrying classes of chemicals. Modern immunochemical, chromatographic, and spectroscopic systems exploited to analyze AFs are operative; nonetheless, original or developed methods to enhance the speed, detection, and accuracy of AFs determination would lead to safer feed commodities. AFs remain a dominant food safety issue, especially in unindustrialized countries where regulatory restrictions do not exist or are not compulsory. For instance, some countries in the world have endorsed AFs-related regulations. Despite being an AFs hotspot, the majority of African countries are without AFs regulations. Precise, accurate, affordable, and simple AFs detection means are requisite to apply regulations and measure the effectiveness of AFs control measures. To block or reduce human and animal exposure to AFs, several control and mitigation measures are utilized. These latter include the prevention of pre- and postharvest AF contamination of agricultural products, or their decrease to tolerable levels in contaminated products through removal, degradation, or decontamination, and regularity limits. In this line, good postharvest handling practices, good agricultural practices, and good manufacturing practices of AFs preventive procedures should be well developed. Scientific progress has also legalized the use of sophisticated biological, physical, and chemical processes for the prevention and decontamination of already contaminated feed matrices. For supplementary investigations, it would be exciting to recognize the importance of the so-called masked mycotoxins to the overall contamination of feed; nonetheless, this might be difficult, as these compounds cannot be detected with conventional analytical procedures.

## Figures and Tables

**Table 1:** Occurrence of AFs in animal feed in some countries from different continents.

Continent	Country	Aflatoxins	No. of Samples	Positive Samples (%)	Range (µg/Kg)	Method	Reference
Asia	China	AFB1	2083	33.9	0.6–1818.0	HPLC/FLD	[114]
			3500	81.9–100	1.2–27.4	HPLC/FLD	[115]
	Pakistan	AFB1	193	65.3	LOD to 180.5	HPLC/FLD	[116]
	Philippines	AFs	107	50.5	<3–1663	ELISA	[117]
Africa	Tunisia	AFB1	136	60.9	17–37.8	UHPLCMS/MS	[118]
	Ghana	AFs	60	100	5.32–29.88	HPLC/FLD	[119]
	Nigeria	AFB1	10	100	1.48–15.50	TLC	[120]
	Ethiopia	AFs	100	96	LOD to 306.9	ELISA	[71]
North America	Mexico	AFs	170	100	25.9–27.0	HPLC/FLD	[88]
Europe	Spain	AFs	193	34.7	0.05–6.45	LC/FLD	[121]
			10	n.d. ^1^	n.d. ^1^	HPLC/FLD	[122]

^1^ n.d.: not detected.

**Table 2:** Emerging detection chromatographic techniques used to analyze AFs in feeds.

Country	Sample	Extraction and Clean up	Detection	Analytes	LOD Range	LOQ Range	Validation Parameters	References
India	Rice	Phosphate-buffered saline, loaded on IAC: (AFLAOCHRAPREP^®^) and eluted with MeOH	UHPLC-FLD	AFs (B1, B2, G1 and G2)	0.02 ng/g	0.25–1 ng/g	Linearity, sensitivity, accuracy and precision	[129]
India	Peanut-processed products and cereal (rice, corn, millets)	IAC and MeOH	UHPLC-FLD	AFs	-	-AFB1, AFG1: 0.025 mg/kg -AFB2, AFG2: 0.01 mg/kg	Selectivity, linearity, recovery and precision	[130]
India	Pigeon pea husk, poultry feed and wheat bran feed	IAC and MeOH	UHPLC-FLD	AFs	-	0.5–2 ng/g	Linearity, sensitivity, accuracy and precision.	[117]
China	Chili powder, green bean, and black sesame	IAC	UHPLC-FLD	AFs	0.1–0.25 ng/g	-	Linearity, accuracy and precision	[95]
China	Corn and wheat	Solid-phase extraction (SPE)	LC-FLD	AFs	0.04–25 μg/kg	0.11–0.61 μg/kg	Recovery and repeatability	[131]
Ethiopia	Maize, wheat bran, dairy feeds	MeCN	ELISA (Optical density)	AFB1 and AFM1	-	-	Recovery and coefficient of variation (%CV)	[132]
Iran	Corn silage; corn; wheat; soybean nutrients dairy cow	MeOH	ELISA (450 nm)	AFB1	-	-	-	[133]
Pakistan	Wheat	Easi-Extract^®^ AF IAC	HPLC	AFs	0.028–0.091 µg/kg	0.066–0.273 µg/kg	Recovery	[124]
Iran	Corn silage	C18 SPE Column and MeOH	HPLC–FLD	AFs	0.015–0.12 µg/kg	0.05–0.4 µg/kg	Accuracy	[125]
China	Maize; wheat; animal feed	AokinImmunoClean CF AFLA	HPLC	AFB1	0.5 µg/kg	1.5 µg/kg	Recovery	[134]
Brazil	Soybean kernels	IAC AflaStarTM Fit	HPLC-FLD	AFB1	0.13 µg/kg	0.37 µg/kg	Selectivity, linearity, accuracy and precision	[135]
Macedonia	Maize; dairy cow feed	AflaPrep^®^ IAC SPE	HPLC–FLD	AFB1	0.005 µg/kg	0.014 µg/kg	Linearity, decision limits (CCα), CCβ, precision (repeatability), and recovery	[136]
Republic of Korea	Corn matrices	AflaTest^®^ WB IAC	HPLC-FLD	AFs	0.01–0.17 µg/kg	0.01–0.51 µg/kg	Linearity, accuracy, and precision	[137]
Turkey	Dairy cow feeds	AflaTest^®^ IAC	HPLC-FLD	AFs	0.046–0.059 µg/kg	0.153–0.197 µg/kg	Selectivity, recovery and precision	[99]
Brazil	Dairy cattle feed	AflaTest^®^ IAC	HPLC	AFB1	-	-	-	[138]
Serbia	Maize	MycoSep^®^224AflaZon SPE	HPLC-FLD	AFs	0.2–0.6 µg/kg	0.6–1.8 µg/kg	Linearity, recovery, repeatability and reproducibility	[139]
Ghana	Maize and groundnut	-	TLC (EtO_2_-MeOH-H_2_O_2_ (96:3:1) at 365 nm	AFs	341 µg/kg	-	-	[100]
Togo	Maize	-	HPLC	AFB1	0.08 μg/kg	-	-	[140]
Colombia	Corn, rice, and cassava		LC–MS/MS	AFs	-	-	-	[141]
